# Community Member Experiences with Conversation-Based Companion Animal Adoption Policies at a Large, Non-Profit Animal Shelter

**DOI:** 10.3390/ani16213389

**Published:** 2026-10-27

**Authors:** Kaleigh M. O’Reilly, Liana R. Moss, Alexis Bettencourt, Lindsay Hamrick, Kevin N. Morris

**Affiliations:** 1 Institute for Human-Animal Connection, Graduate School of Social Work, University of Denver, Denver, CO 80210, USA; kaleigh.oreilly@du.edu (K.M.O.); liana.moss@du.edu (L.R.M.); alexis.chico.12@gmail.com (A.B.); 2 Humane World for Animals, Washington, DC 20037, USA; lhamrick@nhspca.org

**Keywords:** adoption policy, conversation-based, qualitative, companion animal, phenomenology, adoption returns

## Abstract

Conversation-based companion animal adoption policies have been used by U.S. animal rehoming organizations since the 1990s. These policies differ from traditional adoption approaches that often use screenings or restrictions to determine adopter suitability. Personal accounts indicate that adopter experiences vary significantly depending on the adoption policies an organization uses and therefore should not be assessed collectively. With recent research indicating that conversation-based policies may lead to more equitable access to the human–animal bond, there is a need to understand how these specific policies are experienced by adopters. This study used a phenomenological approach to understand how adopters perceive conversation-based adoption policies. The researchers conducted semi-structured interviews with 30 community members at the Wisconsin Humane Society Milwaukee Campus. Participants described a consistent adoption experience, highlighting strengths, aspects for improvement, and how they view such policies affecting their community. Moreover, participants identified key aspects of the organization’s adoption policies that both allow for and could be expanded to create more equitable access to the human–animal bond. Animal shelters and rescues should use these findings to optimize community participation in adoption programs and advance animal welfare.

## Simple Summary

Current literature indicates that companion animal adopters’ experiences differ significantly depending on the policies of the organization they are adopting from. Given recent research suggesting that conversation-based adoption policies may lead to more equitable access to the human–animal bond, this study used semi-structured interviews to understand how adopters experience these policies, what mechanisms may be creating greater access, and how such policies could be expanded. Animal shelters and rescues should use these findings to optimize community participation in their adoption programs.

## 1. Introduction

Animal adoption (hereafter referred to as adoption) is central to companion animal (hereafter referred to as animal) welfare efforts because it facilitates the transition of currently unowned animals from the shelter environment into permanent homes. The successful rehoming of unowned animals into a healthy environment is fundamental to the mission of animal shelter and rescue organizations that are dedicated to minimizing the euthanasia of healthy animals (while both shelters and rescues serve stray or unowned companion animals in the U.S., they differ in physical facilities, municipal vs. private/nonprofit status, intake obligations and discretion, role in the sheltering system, and funding sources) [1,2]. Formal adoption programs and policies are key to reaching this goal, but their effectiveness relies on public involvement. Negative community views of shelter adoption policies can result in fewer successful adoptions [1]. Therefore, understanding community perspectives is essential to designing and implementing effective adoption programming. Community engagement research demonstrates that incorporating the experiences and feedback of those served by an organization can help identify barriers, improve accessibility, strengthen trust, and create programs that better meet community needs [3,4]. Likewise, research examining how communities perceive animal adoption practices is necessary to improve shelter–community relationships and advance animal welfare.

Adoption policies utilized by animal shelters and rescues are typically of two main categories: traditional (‘closed’) and conversation-based (‘open’). Traditional adoption policies have been known to discriminate against socially and economically marginalized populations due to arbitrary requirements and burdensome processes [5,6,7]. Conversation-based policies, such as those detailed in Humane World for Animal’s Adopters Welcome training program [8], specifically remove unnecessary requirements and obstacles. These types of policies prioritize matching adopters with animals based on compatibility through informed conversations with shelter staff or volunteers and the belief that not every home that is good for an animal will look the same [9]. Adopters Welcome training is guided by the philosophy that a deep connection with pets transcends socio-economic, racial, and geographic boundaries, and that no one should be denied the opportunity to experience the benefits that come from the human–animal bond. Research indicates that those who adopt under conversation-based policies form strong connections and provide a similar level of care to that of adopters who acquire animals through traditional screening-based adoption policies [10], and that when an animal shelter shifts to conversation-based policies, they can engage more marginalized communities in adoption without an increase in animals returned to the shelter [7,11].

Only a few qualitative publications have focused on exploring the adoption experience across a variety of adoption programs [5,6,9]. These studies suggest that adopting an animal from a shelter or rescue is not a homogeneous process, but rather, one’s experience varies according to the policies and procedures of the organization and/or a particular identity group a potential adopter belongs to (e.g., socioeconomic status, housing status, race/ethnicity). For example, individuals have viewed organizations that employ traditional adoption policies as being invasive, demanding, and inaccessible to those with lower incomes [6,9,12,13]. Those who have been denied adoption under stringent policies expressed experiencing emotional turmoil, feeling that they were not “good enough” to care for an animal, and were ultimately deterred from adopting altogether [6,9]. These experiences are likely due to traditional policies using more restrictive screening practices that incidentally exclude certain groups of individuals who both want and are able to provide a good home for an animal [9]. Alternatively, individuals have viewed adoption under conversation-based policies as less restrictive and appreciated the general ease of the process, which is likely due to such policies prioritizing reduced adoption fees, having few or no strict requirements, and allowing for same-day adoptions [6]. These differences demonstrate that adoption experiences can vary significantly across organizations with different adoption policies, and therefore, such experiences should not be assessed collectively.

While the findings across previous qualitative studies of adopter experiences are important and include critical community perspectives on different adoption approaches, there is a lack of research intentionally focused on understanding the experience of individuals who specifically adopt under conversation-based adoption policies [5]. With increasing recognition that a shift towards conversation-based adoption can elicit more equitable access to the human–animal bond across the community a shelter serves [7], community members’ perspectives on such policies are critical to understanding how this kind of programming is experienced by the community being served. Furthermore, while conversation-based adoption practices may increase engagement in adoption among marginalized communities when barriers are removed [7], more subtle barriers can persist. By synthesizing the perspectives of individuals who have gone through the adoption process at a shelter that employs conversation-based adoption policies, researchers can better understand why and how these policies generate more adoption engagement. The findings can then inform how such policies and training programs, like Adopters Welcome, can be optimized to expand adoption accessibility to all communities.

The Wisconsin Humane Society (WHS) has been employing the full suite of Adopters Welcome open adoption policies since 2014 and has seen increased access to adoption across the community it serves as a result [7]. For this study, we partnered with the WHS Milwaukee Campus to understand how its adoption program is perceived by members of its community. Thirty participants from the Milwaukee metropolitan area were recruited to participate in the study through semi-structured interviews. Participants had to have either adopted an animal from WHS, adopted and returned an animal to WHS, or engaged with the adoption process at WHS but ultimately not have adopted between 2023 and 2024 at the WHS Milwaukee Campus. These adopters’ perspectives are critical to understanding why conversation-based adoption policies may be leading to the measured increase in access to adoption [7], and how such policies might be optimized to advance the adoption goals central to the mission of animal welfare.

## 2. Materials and Methods

### 2.1. Design

Applying hermeneutical phenomenology underpinned by Gadamer’s philosophy, this study was designed to understand how conversation-based adoption polices at the WHS Milwaukee Campus are viewed by members of the community they serve through 30, one-hour interviews. In phenomenological research, individuals’ lived experiences are used to generate a common meaning of a phenomenon [14]. Using elicited first-person narratives, hermeneutic phenomenology is a circular analytical process in which a researcher’s understanding of the phenomenon is enriched through numerous interactions with the study data [15,16]. Gadamer’s hermeneutical phenomenology is a subset of hermeneutics that is helpful when limited research on a subject exists as it can “reveal the unseen world of lived experiences” [15,17]. The method involves: (1) the researcher(s) acknowledging their current understanding of a subject before engaging in the work; (2) the encounter of a different “horizon”, or perception, through the data collection; and (3) achieving a “fusion of horizon” when the data enlarge the initial understandings of the researcher(s) [15]. For this study, prior to conducting data collection, the research team collectively reflected on their own lived experiences with, and perceptions of open adoption programs. Researchers continued to reflect on how well their perspectives aligned with those shared by the study participants throughout data collection and analysis [18]. To more intimately understand the experiences of the participants in this study, the researchers utilized their multidisciplinary background (e.g., shelter program manager, social work, community engagement) with knowledge of, and lived experience with, a variety of different types of animal shelter adoption programs and community engagement initiatives [17,19].

Individuals were eligible to participate in this study if they had engaged with WHS in the last two years (2023–2024) in the capacity of either having adopted an animal, adopted and then returned an animal, or went through the adoption process but ultimately did not adopt an animal. Acknowledging that returning an animal or being denied adoption of an animal could potentially be sensitive topics [9], research procedures were evaluated prior to data collection for alignment with trauma-informed principles in an effort to minimize physiological and psychological harm for both the participants and researchers [20]. This process involved researchers acknowledging their positionality in relation to the research and participants, as well as rigorously evaluating the interview questions’ ability to affirm the lived experience of WHS community members. Moreover, researchers took time to build rapport with the research participants while facilitating teach-back consent, and they emphasized the participant’s right to decline to answer any questions or withdraw from the study at any time.

### 2.2. Recruitment

Recruitment for the study was conducted under a University of Denver Institutional Review Board-approved protocol (DU IRB Protocol #2232163-1). Researchers partnered with staff members at WHS to recruit study participants. Recruitment flyers were posted in visible areas inside the Milwaukee Campus, and recruitment emails were sent out to all individuals who had gone through the adoption process at the WHS Milwaukee Campus in the last two years by shelter staff. Shelter staff only provided study information to potential participants and were not involved in any other aspect of recruitment for the study. Recruitment materials contained information about the research study, a link to an opt-in form, and contact information for the principal investigator so interested parties could ask questions before completing the opt-in form. Both recruitment materials and the opt-in form were made available in English and Spanish. Items in the opt-in survey included the consent form, basic demographic questions (race, ethnicity, age group, disability status, housing status, and preferred language), contact information, and study-specific questions, such as geographic proximity to the shelter, species of animal adopted, and adoption status. The latter collected information on the outcome of the individual’s interaction with the adoption program as successfully “adopted”, “adopted & returned”, or went through the process but ultimately “did not adopt”. Electronically signed participant consent forms were stored in REDCap, a secure data management system hosted by the University of Denver [21,22].

One-hundred and four individual WHS community members responded to the initial opt-in survey. The demographic information was used by the research team to assess community members’ eligibility to participate in the study. Eligibility included being at least 18 years old; having engaged with the WHS Milwaukee Campus adoption process in the last two years; living within 25 miles of the WHS Milwaukee Campus; and consent to being audio-recorded. Of the 104 respondents, 96 met the inclusion criteria for interview participation. Demographic information from the opt-in survey was also used to select a diverse sample of 30 WHS community members to participate in the interviews. A “diverse” sample was defined as having broad representation across: age, gender, race, ethnicity, housing type, disability, preferred language, and adoption status. After creating the primary study sample of 30, we selected an additional 20 respondents, with the same attention to diversity, for a waitlist in case someone from the original study group could not be contacted or were not able to show up for their interview.

The primary sample of 30 individuals were contacted up to three times via their preferred contact method (email, text, or phone call) to invite them to participate in a qualitative interview. At least three days elapsed between contact attempts. If an additional contact method was provided, it was used in addition to their preferred method during the second and third contact attempts. If an individual did not respond to three contact attempts, or indicated that they were no longer interested in participating, an additional person was pulled from the waitlist in order, and contact was attempted in the same fashion. Similarly, if an individual failed to show up for a scheduled interview, we first attempted to reschedule before contacting someone from the waitlist to fill the spot. With this method, all 30 interview slots were ultimately scheduled and completed.

### 2.3. Data Collection

One-hour, semi-structured interviews were completed with all 30 consented study participants between February 2025–April 2025. Most interviews (*n* = 22) took place in-person at the WHS Milwaukee Campus, with two conducted over Zoom video conferencing (Zoom Video Communication, Inc., San Jose, CA, USA), during the week of February 24th between the hours of 11:00 a.m.–7:00 p.m. Central Time. We conducted six additional interviews in March and April via Zoom video conferencing because participants needed to reschedule or alternates needed to be contacted from the waitlist. To support the hermeneutic process, interview questions were designed to encourage each participant to reflect openly on their experiences with the conversation-based adoption process at WHS [19]. In addition to gathering data about participant experiences with the WHS adoption process, several questions focused participant reflections on how this type of adoption program impacts their community, their experience with other animal adoption programs, and suggestions for things to add, remove, or improve upon to create a more equitable animal adoption program and experience for their community (Appendix A). After completing the interview, participants received a $100 Visa gift card as compensation for their time. Interviews were audio-recorded, de-identified, and stored in a password-protected database. A second-party vendor (GoTranscript, Ltd., Lewes, DE, USA) transcribed the de-identified audio recordings. To ensure the accuracy of the transcripts, each transcript was reviewed by a member of the research team, and any discrepancies found were corrected. Verified transcripts then underwent data analysis.

### 2.4. Data Analysis

As there exist no formal recommendations for utilizing Gadamer’s hermeneutical phenomenology in qualitative analysis, other researchers with expertise in phenomenological qualitative research were consulted to inform the approach in this study. To achieve the most accurate and relevant account of the topic of study, the researchers were required to regularly acknowledge their own prejudices and stay open to differing perspectives shared by participants so that through consistent dialogue, “the horizon of the interpreter and the phenomenon being studied are combined together” [18]. The memoing process and codebook construction were guided by the philosophical foundations of Gadamer’s hermeneutical phenomenology, primarily through regular group discussions and reflection amongst the researchers. The data analysis process proceeded as follows: (1) researchers immersed themselves in each individual’s experience by reading through each of the transcripts; (2) interpretive memos, or summaries, were generated about each of the major topics that arose in each interview; (3) researchers convened to discuss and refine common themes across all the interviews; (4) finalized themes were entered as codes into a qualitative coding software (Atlas.ti, version 26, Berlin, Germany) to efficiently facilitate the process of grouping and aggregating themes; (5) researchers applied the codes to each interview transcript in two rounds with different researchers coding the same manuscript. This allows for further investigation into experiential differences between individual participants and the checking and reduction in researcher bias and prejudice; (6) all participants in the study were sent an email containing a secure link to a survey where they had the option to anonymously review a summary of the themes and share if there was anything shared in the description of the findings that they would change or add to better reflect their lived experience; several additional open-ended questions on the implications of the findings were also included; (7) feedback received from participants was incorporated into the finalized interpretation of the results reported here. The feedback received from participants was markedly supportive of the interpretation of the results. It is in this final step, the sustained dialogue with participants and incorporation of their feedback, where the hermeneutic circle is taken a step further under Gadamer’s philosophy [18].

### 2.5. Validity and Trustworthiness

Researchers in this study facilitated the hermeneutic circle by critically assessing interpretations of the data and engaging in regular debrief sessions to challenge preconceived assumptions and biases regarding the phenomenon [15]. The findings reported in this study incorporated direct quotes from participant interviews whenever possible to engage the reader as an additional check on the validity of the data interpretations [18]. Nine participants (30%) gave feedback on the summary of findings and answered the open-ended questions in the optional survey. While this is a somewhat low response rate, researchers did not have major concerns as member-checking is not essential to closing the hermeneutic circle in phenomenology [19].

## 3. Results

### 3.1. Demographics

Of the 30 participants (Table 1), 19 (63%) self-identified as female, 8 (27%) as male, and 3 (10%) as non-binary. Regarding race, 2 (7%) participants self-identified as Black, 2 (7%) as Asian, 23 (77%) as White, 4 (13%) as Hispanic, and 1 (3%) as American Indian. Participants were somewhat evenly spread across age groups with 9 (30%) self-identifying as between the ages of 18–30, 6 (20%) as between the ages of 31–45, 8 (27%) as between the ages of 46–64, and 7 (23%) as 65 years of age or older. There were 17 (57%) participants who self-identified as renters, 11 (37%) as homeowners, 1 (3%) as being in temporary housing accommodations, and 1 (3%) as having some ‘Other’ type of housing status. In response to disability status, 12 (40%) participants self-identified as living with a disability, with 9 (30%) of those individuals describing their disability as invisible, and 3 (10%) describing their disability as both visible and invisible.

Participants were asked to identify their adoption experience(s) at the WHS Milwaukee Campus as fitting into one or more of three categories. Of the 30 participants, 23 (77%) self-identified as having adopted an animal during their experience, 6 (20%) as having adopted and then returned the animal to WHS, and 4 (13%) as having gone through the adoption process but ultimately not adopting an animal. The majority of participants, 21 (70%), had adopted or looked to adopt a cat, 12 (40%) participants had adopted or looked to adopt a dog, and 1 (3%) participant had adopted another species of animal.

While all participants in the study were required to have adopted, adopted and returned, or attempted to adopt at least one animal from WHS within the past two years (2023–2024), 12 (40%) individuals indicated in their interview that they had previously adopted from WHS at least one other time prior to the study inclusion period, if not multiple times across the last four decades. There were 2 (6.7%) individuals in the study who adopted their first animal from WHS in the early 1980s, 4 (13%) in the 2000s, and 6 (20%) in the 2010s and early 2020s.

### 3.2. Theme 1: Why WHS Milwaukee Campus Was Chosen for Animal Adoption

Participants stated a variety of reasons for choosing the WHS Milwaukee Campus when considering animal adoption. Many noted that the WHS Milwaukee Campus was local to where they lived, or close by, making it an easily accessible option. Multiple other participants discussed how they were generally familiar with the organization because of its reputation in the community, seeing it featured on the local news, or having been referred to the organization by family, friends, or coworkers. Some participants described how they had previously adopted from the organization, with one participant saying that they had been “coming here for animals ever since I could remember (P12).”

***I appreciate the mission […] If I’m gonna donate my money somewhere, it’s gonna be here, versus like—I don’t do breeders (Participant 6)***. The perception of WHS Milwaukee Campus as a “rescue setting” (P2) was attractive to many participants, with one sharing that they themselves are a “big believer in adoption” (P13) and another saying, “I wanted to save an animal if I could” (P1). Similarly, regarding motivation for choosing to go to WHS, several participants indicated that they had previously adopted from other humane societies in the past, with one perceiving this choice as “the most ethical way to go about it” (P24) and several others stating that they would rather financially support an animal shelter or rescue organization over alternative options.

Furthermore, expectations of the overall adoption process and specific adoption policies at WHS motivated many participants in the study to choose WHS to adopt an animal. Several participants discussed that they like adopting animals through WHS because they can have the expectation that the animal they adopt is “at least gonna be fixed” (P6) (i.e., spayed or neutered) and not be sick. “I feel confident in the pets that they have available to people, that they’ve you know, taken their time to make sure they’re healthy and get them neutered and spayed” (P23). Other participants spoke to the adoption process broadly, describing how they like that WHS works with potential adopters to find the animal that is the best “match within your household” (P27). Several participants pointed directly to marketing strategies and adoption-specific policies when reflecting on why they chose to go to WHS:

*I like their bench warmers, their color-coded status […] to kind of denote the dog’s energy level, but the fact that cats over a year [old] are, ‘Pay what you want to, just make a contribution’, that was a really big deal*.

(Participant 16)

An animal that has been donated as a “bench warmer” has been waiting longer than average in the shelter to be adopted. The “pay what you can” option for cats over a year old was a WHS policy highlighted by multiple participants as their reason for choosing WHS when adopting an animal, with one participant noting that this policy in particular makes adoption affordable for them.

### 3.3. Theme 2: The Conversation-Based Adoption Experience at WHS

***The experience is always the same. It was a good experience. Usually, when you sign up for the waitlist, you put the pet that you’re looking for if you have an idea of a pet. They get to you when they get to you and it’s on a case-by-case basis (Participant 6).*** Participants described a consistent adoption process experience at the WHS Milwaukee Campus. Before starting the adoption process, information about animals available for adoption could be viewed on WHS’s website. Potential adoptees began by signing in at the front desk and joining the waitlist to meet with an adoption counselor; they could also list what animal(s) they were interested in meeting. Some participants said that they were able to join the waitlist before arriving at the facility through an online queue via the website. The waitlist was “first come, first serve” and participants described waiting anywhere from a few minutes up to 6 h. Some individuals mentioned that on busier days, there was a limitation on how many animals they could meet. Once an adoption counselor was available, participants were typically guided into a private room where they could meet the animal(s). If the animal a participant was interested in was unavailable, or not a good fit, the adoption counselor would try to find a compatible match. During this time, potential adoptees could ask any questions about the animal, and the adoption counselor shared all known information, including observed behavior, health history, and origin. If a participant decided to adopt, they would complete the adoption forms distributed by the staff. Adoption fees for cats over a year old were “pay what you can”. A few participants mentioned receiving brochures on basic pet care, a free 30-day pet insurance trial, and local veterinary recommendations, as well as veterinary visit vouchers for the animal’s first appointment. Other participants stated that they had also received a starter pack of essential pet supplies, such as a carrier, pet food, blanket, and/or a leash. Only 4 participants interviewed stated they “Did Not Adopt” from WHS. These participants shared that they felt the animals they wanted to adopt would not be a good fit in their homes. No participants stated that the adoption process at WHS was the reason why they did not adopt.

A few participants adopted animals through foster homes associated with the WHS Milwaukee Campus. Participants contacted foster volunteers directly to schedule a private appointment to meet the animal they were interested in, which often took place at the foster volunteer’s private residence. If they decided to adopt, participants reported meeting the foster volunteer and their future pet at the WHS facility for a final, private meeting with the animal, and then filled out the necessary adoption paperwork. After the paperwork was completed, individuals were allowed to take their new pet home.

Many participants in this study had been adopting animals through WHS for several decades and had witnessed the shift in the adoption process after the implementation of conversation-based practices. Multiple participants reported experiencing a stricter adoption process in the past. For example, a few participants shared that in past adoptions (10 or more years earlier), WHS conducted landlord verifications, in which a renter’s landlord was contacted to confirm the lease allowed for a pet, and that there was a limit of two animals they could meet with during adoption counseling. Recurring patrons noticed that over time, WHS had a stronger focus on compatibility compared to their previous experience, as the organization used to be, “more concerned with the mechanics of the adoption rather than the counseling aspect” (P9).

#### 3.3.1. Theme 2a: Adoption Process Strengths

Participants shared a variety of different aspects of the adoption process at WHS Milwaukee Campus that they considered strengths. The majority highlighted the ease of the adoption process and considered it “a great experience”. Many participants pointed to the online experience as useful for a number of reasons, including that the website was consistently updated with the animals currently available for adoption, it allowed for completing the pre-adoption questionnaire, and gave participants the ability to “join the queue” to see an adoption counselor directly from the website before arriving at the shelter. Furthermore, many participants felt that a strength of the adoption process was that WHS had a good selection of animals, and that adoption counselors allowed them to meet as many animals as possible and spend as much time with the animals as needed prior to deciding on adoption. Participant 6 shared, “I do like that they give you time to spend with the animals, so that you can get to know them.” Another participant said, “it is nice to be able to walk around and see all the animals, ‘cause not every shelter you can do that.” (P9).

***[Adoption counselors] do a really good job of trying to match up the right pet for the right person (Participant 16).*** Many participants shared that their adoption counselor contributed to the strengths of the adoption process. Adoption counselors were knowledgeable about the animals, they helped ensure the animal was the “right fit”, and did not pressure participants into making an adoption decision.

Several participants suggested a strength of the process was receiving a set of resources to get started with their animal, such as a “litter box, litter, bag of food, toy, and brush” (P28). Participant 29 was appreciative of the information they were given during the adoption of their dog:

*I hadn’t had a young dog for a while. So, it’s nice to get that information about a different animal, the younger animal. Those are really, really great tools. And it’s really helpful for people to have that information right at their fingertips, you know, a number to call the behaviorist*.

(Participant 29)

Participants also spoke about WHS’s “pay what you can” for cats older than one years as a strength of the adoption process. Two participants shared that they did not know about WHS’s “pay what you can” prior to adopting their cat and chose to donate what would have been their payment since, as Participant 8 expressed, “I was expecting to pay, so I just paid what I had.” Participant 6 shared, “I always just make a donation since I don’t have to pay for my cat.”

#### 3.3.2. Theme 2b: Adoption Process Challenges

***The downfall was that we waited there all day. It was apparently a very popular day to get [adopt] animals (Participant 4).*** The majority of participants felt that they experienced no challenges with the adoption process. Only a few participants described encountering some challenges with the adoption process, with the most cited challenge being that wait times to meet with a counselor could be unpredictable. Wait times were described as variable depending on the day of the week, with weekends being the busiest days plagued by longer wait times. The process of meeting the animals was considered overwhelming by some because of the setting. Participant 14 shared that they felt meeting animals was more stressful on busier days because, “it’s hard to find a quiet space to do that”, and a few participants mentioned feeling “rushed” after deciding to adopt an animal. One individual felt that they were misled by their adoption counselor because the animal they adopted exhibited behaviors in the home they hadn’t been made aware of, and there was also some confusion regarding the online process, with a couple of participants sharing that the QR code check-in and wait list queue were challenging to figure out upon arrival at the shelter. Participant 24, a participant who had a foster-based adoption experience, shared:

*I think one of the drawbacks of that [adopting through a foster] is, it is a little uncomfortable communicating with a stranger […] So feeling like, ‘Do I have to make a decision there in the moment?’ The foster mom that we worked with was really great. She said, ‘You don’t have to tell me an answer today, you can come back.’ And we ended up coming back the next day. I would say that that’s kind of maybe uncomfy, but that’s also just a part of the foster process*.

(Participant 24)

Most participants felt that the information shared by adoption counselors about the animals they were interested in adopting “was quite comprehensive” (P23), and that all information that WHS had about an animal was shared with them prior to an adoption decision. However, a couple of participants expressed that there was some unavailable information about animals, but it was generally understood that WHS would have no way of knowing the information they wanted: “If they don’t have it, they don’t have it, right? So it’s hard for them to answer a question that they don’t have the answer to” (P29). On the other hand, a few participants expressed that while the information shared about animals was sufficient, they felt additional guidance on the behaviors and basic needs of the animals, including preventive care (heartworm, flea/tick medication), would have been helpful.

#### 3.3.3. Theme 2c: Staff Treatment of Adopters

Participants reported similar treatment from and interactions with WHS staff and volunteers, with the majority describing these experiences as friendly and welcoming. However, individual participant experiences tended to vary based on how busy WHS was during the time they visited. The staff at the front desk were described as patient and communicative by most participants, with one detailing that:

*[Front desk staff] did what they could do in a setting with a lot of impatient people […] keeping everybody updated and checking in and giving the kids coloring sheets and anything they could do to make it a good experience*.

(Participant 4)

During high visit volume times at the humane society, some participants described that the staff shared inaccurate wait times, and Participant 7 noticed that some volunteers stationed at the welcome desk struggled to deescalate customers who were unhappy about wait times. Regarding adoption counselors specifically, most participants characterized them as “outgoing and patient” (P3). A couple of participants noticed during busy times that their adoption counselor seemed “overwhelmed”, but they were reportedly still able to be “accommodating” (P4) and remain professional, as expressed by Participant 17:

*She [the adoption counselor] was kind of done at that point, like when you’re doing a job, but everybody calls in sick, all this has to be done, but it’s now just you to do it […] but she, at no point in time, was rude or anything […] no time did she come across that way. She said, ‘Hey, we’ve been shorthanded. I’m so sorry. It’s been a long day.’ But she still took her time and that meant a lot*.

(Participant 17)

***I didn’t feel like anybody was getting preferential treatment…everybody was being treated the same (Participant 30).*** A vast majority of participants described the staff’s treatment as fair and respectful throughout the adoption and return experiences. Participant 24 also noted this fair treatment during their experience with the foster program and described the interaction as “open and transparent”. Many shared a similar testament, as detailed by one participant: “It was overall, just a really good experience [with the staff]. I wouldn’t say there was anything negative about the way the adoption staff had treated us” (P21). Adoption counselors were repeatedly described as understanding by numerous participants. Specifically, Participant 26 expressed gratitude that their adoption counselor was respectful about their decision to ultimately not adopt and shared that the counselor mentioned other animal shelters in the area that may have an animal who would be a good fit for their situation.

While no participants described feeling that they were treated in an unfair or disrespectful manner by staff, a few participants described feeling “confused” about the service queue, and that it felt “unfair” because it appeared that some potential adoptees were being serviced out of order, and Participant 8 described having an “awkward” interaction with a volunteer regarding a misunderstanding about a discount policy on pet supplies from the shelter’s store.

#### 3.3.4. Theme 2d: Questions and Conversations During the Adoption Process

Participants described general questions asked during the adoption process at WHS Milwaukee Campus as minimal and “nothing super personal” (P1). Participants filled out a short questionnaire online prior to adoption, which included questions about animals the participants had previously adopted, if the participant had adopted and then later returned, if children lived or frequented the residence (and how old), and some optional demographics about the participant such as age, race, and current address. Participants stated that no follow-up questions regarding the questionnaire were asked upon arrival at the facility, but that there were some additional questions asked by the adoption counselor once the adoption process commenced.

***I’ve never had them ask me any probing questions about if I can afford to take care, or like…when I got a cat, they never asked me, ‘Are you able to provide annual vaccines?’*** (Participant 6). The majority of participants reported that they were not asked to describe their financial situation to adoption counselors; instead, questions focused on what they were looking for in an animal, such as desired size, activity level, and general concerns. A few participants reported discussing with a counselor why they wanted to adopt, past pet experience, and current pet supplies. Participants with other pets already at home, had conversations with staff about those animals, often discussing their size and temperament as well as a plan for introduction. Some participants experienced staff asking them about their work schedules. A few participants could not remember specific questions they were asked by the staff, but did emphasize the general nature of questions during the adoption process:

*Other rescues sometimes ask way too many questions, and have so many restrictions for animals that sometimes the right house doesn’t meet all of those. Here, they ask a good amount of questions before you adopt, but it’s not crazy like you need a fenced-in yard or like you need all this extra stuff*.

(Participant 7)

“I wouldn’t say there was any information that I felt like I couldn’t share” (P18). The majority of participants indicated that no questions asked during the adoption process made them uncomfortable. However, Participant 28 mentioned discomfort when a staff member asked about their work schedule and felt “guilty” after revealing how many hours they were away from home. Another participant expressed being afraid that their application would be rejected after they had to inform WHS about a previous pet they had adopted and then returned, but that adoption staff, “were understanding once I was able to explain what was going on” (P7).

Only a couple of participants expressed discomfort towards some questions included in the online questionnaire. Participant 26 felt uneasy disclosing the species of their existing pet. They feared WHS would prevent them from adopting due to negative public perception about owning reptiles. Another participant felt awkward about sharing details about other animals living in the residence at that time, stating that, “our living situation was kind of hard to describe online” (P7). This issue was later resolved when the participant was able to talk to a staff member in person and describe their unique situation. Participant 8 felt uncomfortable when asked about the children living at the residence in the online questionnaire. They were concerned about how the information on their child’s age would be perceived by staff and were fearful they would not be allowed to adopt. Another participant was anxious about detailing the number of animals in their residence, specifically because they worried their answers would prompt a staff member at WHS to contact their landlord:

*My apartment only limits two cats per family, and we have more than that. There’s always gonna be a slight uncomfortableness answering that question […] but it didn’t stop me from answering truthfully. Just was, fingers crossed that they wouldn’t reach out to our apartment or anything like that*.

(Participant 21)

While most participants appreciated the short questionnaire and the general questions asked during the counseling process, a few participants suggested increasing the number of questions to vet potential adoptees. For example, Participant 6 proposed that WHS should incorporate questions about affordability and discuss the annual cost associated with caring for an animal with potential adoptees.

### 3.4. Theme 3: The Return Experience

The participants in this study who returned the animal they adopted all reported needing to do so because it was “not a good match” for their family, most often due to behavior with other pets already in the home or their lifestyle. Participants who indicated they had adopted and then returned an animal at WHS described a similar general return process. This involved making an appointment by phone to bring the animal back, answering some questions during the appointment, filling out a form about the animal’s observed behaviors, and saying goodbye to the animal in a private room. Some participants described having to pay a fee to return the animal they adopted if the return took place seven or more days post-adoption.

A couple of participants mentioned that the return process was “confusing”, with one describing the challenge of having to wait several weeks to bring the animal back due to the shelter’s current capacity. One of the biggest challenges shared by most participants, as it related to returning an animal, involved internal, personal feelings of “shame” and “guilt” stemming from not being able to keep the animal. This is exemplified in the following quote from Participant 24:

*One of the things that—having to return two cats, is there’s a lot of shame involved with that, a lot of guilt and lot of like, I don’t want to be perceived as somebody who is flippant about taking an animal home and then being like, ‘Oh, I don’t like them.’ And, that wasn’t the case. Just not great fits with our other cats. It was just—so heartbreaking. My partner and I are still in so much a wreck about it*.

(Participant 24)

***They [shelter staff] were gentle, they were kind, they understood (Participant 29).*** An important distinction is that participants were clear that the staff involved in the return process did not contribute to these feelings, with one participant saying, “I do remember being shocked that I wasn’t being shamed or pressured or anything like that” (P4). Other participants described the staff as nice, gentle, and kind. Participants shared that the staff were “very reassuring” and consoled them, saying things such as, “It wasn’t the right fit. No harm. You’re okay. It’s fine. We’re not angry” (P29), and, “If anything, he [the animal] got outta here for a week, and we know more about him. We have information that’ll help place him in a home that he’ll be good with” (P28). Highlighting further the impact of these kinds of interactions with staff members when going through the process of a return, one participant shared, “Had they shamed me, I think I would’ve been less likely to try [adopting] again” (P4), and another participant stated, “I feel like the staff here would treat me with the same amount of respect, and the same amount of shared excitement of getting a new animal if I were to come back and go through the adoption again” (P24).

Participants in the study perceived potential barriers to returning an animal at WHS. Several participants discussed the fee that is required when returning an animal “after their seven-day grace period”. One participant said that they feel this policy, “discourages people from wanting to try out certain dynamics because I feel like seven days truly isn’t a long enough time to see if an animal is suited for your home or your family” (P13), and another hypothesizing that paying the return fee, “would be really difficult for someone who doesn’t have the money” (P27). Other barriers mentioned by participants included having to make a phone call to schedule an appointment as “there’s a lot of people that don’t like making phone calls” (P24), and potentially having to wait several weeks to return the animal, especially when that animal is not a good fit with other pets in the household.

### 3.5. Theme 4: The Impacts of Conversation-Based Adoption Programs on Communities

***[The adoption process] doesn’t discriminate if you don’t have a lot of money—you can still adopt (Participant 12).*** The majority of participants in this study perceived low barrier adoption policies and practices at WHS to have a positive impact on their community and that this type of program supports more people in their community in gaining animal companionship. Several participants highlighted the “pay what you can” policy for cats over a year old and other regular adoption promotions at WHS as particularly beneficial to their community because “people on limited incomes still have adoption available to them” (P9), and participants believed more families would be encouraged to consider adopting an animal if they did not have to also worry about high fees. Resources received (food, litter, information) upon adopting an animal from WHS were also seen as beneficial to the community since the practice can help lower-income individuals get started with pet adoption. Similarly, adopting animals having already received necessary vaccines and undergone spay or neuter surgery, was a positive for the community because adoptees would not need to budget for the additional costs associated with these veterinary procedures. Numerous participants mentioned that WHS’s less invasive questionnaire during the adoption process was positive for the community because the questions were “reasonable”, making it easier for individuals to complete. Furthermore, WHS’s absence of housing checks and restrictions, not guessing at dog breeds (to protect against landlord breed bias), and focus on genuine compatibility between adoptee and animal were often highlighted by participants who hypothesized that these practices likely led to “a lot more animals ending up in homes” (P25). Moreover, while not an embedded part of the adoption process, many participants pointed out the pet food pantry, which is visible upon walking into WHS, as having a positive impact on their community because it shows adopters a place one could bring extra pet resources they may have and assures new pet owners that after a successful adoption, “if we fall on hard times, we could go get support” (P4).

While many positive impacts of low-barrier adoption practices at WHS were acknowledged by the participants, some identified aspects of the adoption process that had neither a positive nor negative impact on their community. Although WHS provides some information in printouts or pamphlets to new adopters, Participant 28 felt neutral towards this practice because they believed that even more resources should be offered so that adopters do not have to search for these resources independently. They described that this would ease the fear that pet ownership is a lot of work. The free veterinary voucher received upon adoption was viewed as a neutral impact on the community by Participant 22 because they had adopted a cat who had just been visited by the veterinarian in the shelter, so they felt, “it wasn’t really necessary” to use it right away, though it could only be used within two weeks post adoption. Participant 23 expressed that the lack of in-depth questions during the adoption process had a neutral impact on their community, as it is not a “negative impact for individuals”, but was unsure if it was thorough enough to place animals with “good pet parents”.

Participants in this study also shared what they perceived as negative impacts of low-barrier adoption policies and practices on their community. Multiple participants viewed WHS’s “easy” adoption process as having a potential negative impact, since there is a possibility for animals to end up in “the wrong hands”, which “could affect the community, depending on what they’re doing with those animals” (P6). A few participants mentioned the limit on animals viewed per appointment could hinder community members from adoption if they were unable to find the right match within one session, and the variable and unpredictable wait times for appointments were understood by some participants as a negative for their community because “not everybody could sit [in the waiting room] there for hours and hours” (P6). Additionally, a couple of participants stated that WHS’s hours of operation excluded community members who could not work around the facility’s timeframe. One participant felt that the requirement to disclose one’s zip code on WHS’s adoption application negatively impacted their community, as staff could potentially make judgments about whether or not an individual is a good candidate for adoption based on the neighborhood they live in. Moreover, Participant 18 perceived that the online application process could be difficult for community members who lacked access to a reliable computer or were uncomfortable with technology. Some participants highlighted that adoption cost disparities among animals (e.g., a kitten costing $150 versus a cat over a year old being “pay what you can”) could negatively affect community members. Specifically, Participant 9 felt that because of these cost disparities, individuals on limited incomes were now restricted in their choice of pet. Moreover, a couple of participants expressed the ambiguity around additional pet supplies necessary for adoption (e.g., required purchase of a carrier for an oversized cat or medical supplies) as a negative impact on financially strained members of the community. Participant 17 stated that an undisclosed expense could be, “enough of a barrier to not get a pet”, and Participant 11 indicated WHS staff who overly stressed the financial obligations of animals could make certain community members feel as though “they are not good enough” for adoption.

### 3.6. Theme 5: Experience with Other Animal Adoption Processes/Acquisitions

The majority of participants in this study indicated that they had at some point adopted (or attempted to adopt), purchased, or acquired an animal from a source other than the WHS Milwaukee Campus. Many participants adopted animals through another humane society, rescue, local municipal shelter, another WHS campus, or through fosters, home-to-home programs, or “pop-ups” hosted by a shelter or rescue. Other participants indicated that they acquired a pet via family, friends, neighbors, and coworkers, while some found an animal as a stray outside. Multiple participants purchased an animal from sources such as a breeder, pet store, farm, website, and a reptile convention.

There was no consistent experience in animal acquisitions from non-WHS sources described by the participants. Some participants said that their adoption experience at their local municipal animal shelter and other humane societies shared similarities with their experience at WHS Milwaukee Campus. In particular, participants pointed to adoption counselors in other experiences also being “patient” and “wonderful” (P3), adoption being a “quick process but not a rushed process” (P26), and upon completing the adoption receiving things such as print out of information and starter bags of food.

Participants shared a number of differences in their animal adoption experience at WHS compared to other animal acquisitions. Regarding the acquirement of a pet from family or friends, several participants discussed how there could be more information about an animal because they could talk with the previous owner about an animal’s history and behaviors in the home. However, other participants described that there was also often “even less information” (P15) about an animal. This was especially true if the animal was a kitten or puppy. Picking up young animals from friends or family also typically involved having to then obtain extensive veterinary care (vaccines, spay/neuter, flea/tick, microchip) on their own, whereas these procedures/routine care are completed prior to an animal being available for adoption at WHS. This experience was also true for those having found and taken in a lost animal. Furthermore, noticeable differences in animal health stood out for some participants who purchased an animal, with one participant describing a dog they purchased from a breeder as being in “horrible shape” (P25), and another describing how they had to return a kitten bought from a farm the very next day because of how ill they were. Regarding adoption experiences at other shelters, some participants appreciated the ability to make an appointment to adopt, rather than joining an online queue like at WHS, and to put an animal on hold, which gave them time to think about their decision. Other participants were fond of shelters that offered a several-day “trial period” or “test-drive” (P29) program where “you foster the animal for a couple days and then you adopt” (P2). Alternatively, Participant 4 described a shelter that did not allow animals to be returned under any conditions, and when the dog’s behavior turned out to be incompatible with their family, they had to re-home the animal on their own. Several participants also noted having a “warmer welcome” at WHS, noting some experiences at other shelters as less “customer friendly” (P23).

***I think they were using a process of intimidation in the adoption process to get people they wanted. It was like, you know, they were running people through a gauntlet rather than trying to get them paired up with the appropriate pet (Participant 9).*** Multiple participants described adopting an animal from another shelter or rescue as more “intense” (P16), “restrictive, and invasive” (P14), and having to “pass their test” (P20). Several participants recounted having home visits when adopting an animal through a rescue, with one participant describing how they had to submit photos of their home and “proof of a fenced in yard” (P16). Participant 25, remembered not passing a rescue’s initial questionnaire due to lacking a fenced-in yard, said: “It sucked. Because, like, we’re very responsible dog owners, they’re always on leash when they’re outside. So, to be ruled out because we didn’t have a fence was kind of, it felt pretty kind of shitty.” Another older participant chronicled their attempt to adopt from a rescue during which they felt they were denied an adoption by the organization because of their age. As a result, they “went out to a breeder and bought a puppy” (P11). Participant 20 discussed details of an application used by a rescue from which they adopted a dog. The application included extensive questioning about one’s history of surrendering an animal and had clear language explaining that you would be denied adoption if you had ever surrendered an animal. This same application also included intrusive financial questions:

*It made me feel very inadequate and that I wasn’t sure I would be selected, that this was a very choosy process and only the cream of the crop would be allowed. And, that certainly was not my experience here [WHS Milwaukee Campus]. Here I felt like if you have a loving home and you are willing to open your heart and your home, we would like you*.

(Participant 20)

Other participants described adoption experiences at different shelters and rescues as more “tedious” and a “multi-day process”. Participants remembered being required to submit paperwork to their rental office or make a copy of their lease for the organization. One participant described how one shelter called their landlord and also required they set up an interview between their current roommate and the organization. Another participant had to ask family members to fill out character reference questionnaires, while a different participant noted having a “waiting period” while one rescue verified that their other animals had been “properly” cared for by a veterinarian. Participant 10 recounted having to bring their other dog to do a “meet and greet” with the dog they were interested in adopting, describing the process as stressful due to the general environment of the shelter, and that “it was nice having that step eliminated” during their experience at WHS. Participant 17 talked about negotiating with one shelter to adopt out a cat to their daughter, who, while an adult, fell under their 21-year-old age limit, stating that they, “see where they’re coming from ‘cause they want the maturity there. But at the same time, it’s not a one-size-fits-all.” In general, the majority of participants who had a more “restrictive” or “tedious” experience adopting from another shelter or rescue organization indicated they could understand why “high-intensity” screenings and “cumbersome” processes exist, but that “it does negate a lot of people from adopting animals” (P21) who can provide an animal with a good home.

### 3.7. Theme 6: Changes to Conversation-Based Adoption Policies for a More Equitable Experience

***Maybe that would make it more inclusive, having people doing the adoptions [adoption counselors] reflect the community a bit more (Participant 18).*** Participants shared ideas, suggestions, and thoughts on changes that would create more equity in WHS’s adoption process. One participant suggested broadening adoption opportunities to include mobile adoptions to combat transportation barriers some community members might face: “For people who want to have a pet but don’t have transportation, maybe have pop-up locations in different spots that bring some availability to the public instead of them having to come to the campus” (P23). Similarly, Participant 10 suggested having transportation to the shelter available for people with no access to other transportation options. Another suggestion was that WHS should work on increasing diversity within their staff, the types of languages that services are available in, and that when conducting community outreach, WHS would benefit from “having either volunteers or staff who belong to the communities” (P28) they are visiting:

*In various communities, there’s a lot of stigma or ideas about what pets are like, who has certain types of pets and how you should treat your pets. So having people from those communities who can explain, ‘No, you guys, you might really like having a cat.’ And it can fit into your lifestyle. And you know, ‘No, big dogs can be good for kids.’ We just have to find you like the right dog and ‘No, not all pit bulls are terrible.’ It would be nice if the staff reflected all those communities*.

(Participant 28)

Some participants suggested having additional community resources be available like classes or workshops on grooming, nail care and other basic pet information. Participant 28 also shared the idea of state-funded resources: “Honestly, I would have liked state-funded programs, for example, if you are on food stamps, then you also get food stamps for your animal, like litter food stamps, you know. Even better, low-cost, boarding options too.”

Participants also shared recommendations regarding information, pet costs, appointment times, and other topics related to the adoption process at WHS. Participant 20 suggested creating a “cheat sheet” of information for adoption counselors to help them efficiently answer potential adopters’ questions when meeting animals. Several participants felt it was also important to discuss the costs of pet ownership and pet health with adopters, given the low or no-cost adoptions available at WHS.

*They [first time adopters] don’t think about, outside of the food, the bigger picture and how much that could be on the budget. So I think just a mention of annual care, which includes preventatives or something like that, when they’re adopting out, would be beneficial*.

(Participant 6)

A few participants suggested ways to help WHS address wait times and time barriers. Participant 15 proposed using wait times to benefit their adoptions by providing potential adopters with literature about the costs and realities of pet ownership, and Participant 26 thought having two days a week with “extended hours” could allow more flexibility for people and potentially combat busy weekends.

## 4. Discussion

This study is the first of its kind to focus solely on how open or conversation-based adoption policies, as they have been implemented at WHS, are perceived and experienced by community members who have specifically adopted or attempted to adopt an animal. The demographics of participants were well-dispersed across several categories. While a greater majority of participants identified as renters, female and white, there was a roughly even distribution of participants across age group, approximately one-third of participants identified as having a disability, and one-third of participants identified as being non-white and/or Hispanic. Arbitrary adoption requirements, that are often a cornerstone of traditional adoption practices, have been known to systematically discriminate against economically and socially marginalized communities, which tend to have a greater concentration of individuals who are renters, low-income, and/or persons of color [9,23,24]. Traditional adoption policies have also been known to discriminate against both young and older adults as well as those who are differently abled [25,26,27,28,29,30]. Moreover, processes inherent to making adoption decisions in both traditional and conversation-based adoption policies can be influenced by the implicit biases of an adoption counselor [6,9,13,30,31]. Overall, this study found that the conversation-based adoption process at WHS provided a consistent experience between study participants with different identities, housing statuses, abilities, and ages. Study participants recounted adoption decisions being reached collaboratively, with adoption counselors guiding participants as to which animal may be the best fit for a household, and individuals who left the shelter without adopting an animal did not detail being denied an adoption but rather, on the day they were looking to adopt, a “good match” for their household was not able to be found. These findings are important to understanding adopters’ experiences at WHS, however, they do not show that conversation-based policies reduce discrimination in adoptions or improve animal welfare, and these questions require further investigation.

Study participants chose to adopt from WHS for various reasons, many of which are consistent with adopter motivation findings from other studies. For example, the current study found that similar to previous documentation, individuals continue to be motivated to adopt from an animal shelter because they have a strong desire to “save” an animal and/or they believe it is the morally correct choice [9,32]. Furthermore, this study confirms that participants remain motivated by expectations regarding the type of animals a shelter has available for adoption and the medical interventions they have received prior to adoption (e.g., spay/neuter, vaccines) [9]. While previous studies have focused on broad reasons individuals seek to adopt an animal, with our study’s focus on local community members of a single shelter, we also found that geographic accessibility, community reputation, and referrals from family or friends were important motivators to adopt specifically from WHS versus other local options. Another novel finding was that the adoption policies employed by WHS, specifically that they prioritize finding appropriate animal matches for families and that they have regular promotions that reduce overall adoption costs, were motivating factors. This finding is significant, suggesting that some adopters may choose a particular animal sheltering or rescue organization based on its adoption policies, particularly given that potential adopters have been known to avoid certain organizations whose policies impose onerous requirements [9].

The adoption process at WHS was described similarly by all participants in this study, indicating that it did not vary despite individual differences in social identities, housing statuses, stated income levels, and family makeup. Participants praised the ease of the adoption process, which is similar to what participants in other studies have said about adoption processes at shelters with more open policies [9]. Moreover, participants consistently cited working with the adoption counselor as a strength, highlighting their knowledge, flexibility, and efforts to find an animal that was the right fit for their family. The fact that participants pointed out the counselors’ concerns about finding the right match is a critical finding, given that unsupported claims continue to be made that conversation-based adoption policies jeopardize animal welfare by failing to verify “adoptee suitability” with arbitrary adoption criteria [33]. Conversation-based adoptions do verify adopter suitability, but in a way that recognizes that a home and family that is good for an animal has broad definitional ranges [9]. This is further substantiated by the fact that several individuals in this study did not adopt an animal after adoption counseling, citing the reason being that they were unable to find an animal that day that would fit well in their home and with their family. Regarding challenges with the adoption process, most participants did not perceive any part of the process as challenging; however, those who did cited adoption wait times on busy days. While this is not a direct response to the use of conversation-based adoption policies, it is still part of the overall adoption experience and highlights the consideration of additional adoption policies, such as hours of operation and first-come, first-served versus appointments, that can have an impact on how an adoption program is perceived overall. Being able to wait for long periods of time can be associated with societal privilege [34]. Therefore, considering these policies alongside conversation-based policies may help expand access to the human–animal bond through adoption.

While all participants in this study had gone through the adoption process at the WHS Milwaukee Campus, many had previously adopted or attempted to adopt an animal through another animal sheltering or rescue organization. Several participants recounted experiences similar to those at WHS, but many others discussed experiences that differed and described processes and requirements often found in traditional adoption policies [5,6,9,12,13,35,36]. For the differing experiences, words used to describe them (e.g., invasive, restrictive, tedious) were more negative in tone compared to how the WHS process was described (e.g., welcoming, easy, great). Some individuals even shared feeling inadequate about their ability to care for an animal after working with or having their application rejected by an organization that used a more traditional process, despite having already cared for and met the needs of animals in their families. For one participant, after being turned down by a rescue for what they perceived as their older age, they purchased a puppy from a breeder rather than risk being denied again. This study confirms other observations in the literature that individuals’ experiences differ depending on whether they adopted (or attempted to adopt) from an organization that used conversation-based or traditional policies, with those encountering traditional policies more likely to use words with negative connotations in their description [6,9]. These findings are critical for animal shelter and rescue organizations to keep in mind when considering what types of adoption policies they want to employ and the image they want to project. Public engagement in adoption is fundamental to achieving the mission of animal sheltering and rescue organizations [37]. When policies are employed that make it difficult for people to adopt or prevent them from adopting [5], they ultimately harm the broader mission and shrink the prospective pool of homes available to house unowned animals.

Ultimately, with an adoption process that requires adopters to be at the animal shelter to adopt an animal, the interaction with adoption staff is a vulnerable point where inequity can occur. Previous research has found that shelter staff often employ implicit (conscious and unconscious) bias and “gut feelings” to subjectively determine the quality of a potential adopter, and thus who adopts an animal [5,6,38]. This subjective decision-making disproportionately impacts marginalized communities [6,9,36,39]. The Adopters Welcome conversation-based adoption training model may serve as a direct staff-behavior intervention. Quantitative research suggests that this model may engage more marginalized communities in adoption [7], and may reduce discrimination based on family composition and employment status [36]. These findings are supported by the results of this qualitative study of adopters at WHS, who almost universally indicated that they felt they were treated fairly and respectfully by adoption staff. Moreover, no participants in this study shared that they felt discriminated against by adoption staff on the basis of their identities, demographics, or living situation.

Respectful staff treatment is exemplified further in the experiences of adopters who returned their animal to WHS. Previous research has investigated the deeply personal, emotional experience of returning an animal to a shelter, identifying that people often feel a sense of failure, pain, and deep sadness, and that returning the animal remained a distressing memory [40,41,42]. Participants in this study confirmed these findings, reporting emotions of guilt, shame, and heartbreak. In this context, staff treatment and behavior were highly impactful, and importantly, did not compound the emotions the adopters felt when they returned the animal to WHS. Furthermore, these participants shared that given the respect and understanding exemplified by staff, they would be willing and comfortable adopting an animal again. This is an important finding for the field, as shelters and rescues often operate at high volume with a centralized reliance on adopters to decrease their population and reduce euthanasia outcomes for adoptable animals. Returning an animal to a shelter is often highly stigmatized in the animal welfare sector, and in some cases, people have been barred from or shamed into never adopting again [43]. However, research suggests that animal behavior (59%), rather than human personal issues, is the most cited reason for adoption returns [44]. When unrealistic or mismatched expectations have been identified as a return reason, it suggests that poor expectation-setting during adoption counseling, rather than “bad” owners or “bad” animals, may drive such outcomes [45]. The stigmatization of adoption returns is challenged by WHS and the Adopter’s Welcome framework because staff are trained on the potential benefits of time outside the shelter to the animal, including a better understanding of behavior in the home environment and stress relief [46,47]. While this training is reflected in the participants’ recollections of returning an animal, some individuals found other aspects of the process to manifest as frustrating barriers to adoption returns, such as the fee required to return an animal after WHS’s grace period, the phone call required to make an appointment, and extensive wait times. These are potential points of reform for Adopters Welcome programming and important considerations for organizations seeking to incorporate open-adoption policies and practices.

A potential critique of open adoption policies stems from animal welfare concerns. The theory being that policies like fees, veterinarian reference checks, and other forms of screening will protect against impulsive adoption decisions, devaluation, abuse/neglect/cruelty, and relinquishment [5,48]. However, extensive research into the use of screenings and requirements has found that they are rarely indicative of securing significantly greater welfare for adoptable pets compared to open policies [5]. Moreover, an ASPCA study comparing fee-based versus free adoptions of cats found no difference in adopters’ attachment to their cats between groups [48]. The same study asserts that “both groups of cats will likely receive the same quality of care, likely have owners with a similar level of commitment, be equally as likely to stay in the home, and likely be valued equally” [48]. Similarly, a study comparing discounted cat adoption fees with adoptions with a fee of $99 or more found no difference in the retention of adopted cats [49]. In addition, a study of the impact of conversation-based adoption policies at WHS on return rates found that these policies did not increase adoption return rates; adoption return rates actually decreased for cats, and both species saw significantly higher live release rates if they were returned [11].

Unfortunately, the impact of closed adoption policies falls heaviest and most frequently on marginalized communities. Often, these policies are rooted in biased constructs that suggest pet ownership and the benefits of the human–animal bond should be reserved for people who can afford all aspects of pet ownership under all circumstances [39]. In contrast, research has identified that shifting to open-adoption practices can increase adoptions in more marginalized communities, indicating that these policies may be more equitable [7]. Along with equitable access to the benefits of the human–animal bond, community-engaged practice within open-adoption policies, like fee-waived adoption, limiting adoption restrictions, and intentional matchmaking, has had a strong influence on the live release rate of animals in shelters without increasing adoption returns [11,50]. Even so, the results of this study suggest that there are further barriers to adoption that could be addressed to improve equity and animal outcomes. As highlighted by study participants, animal shelters should ensure that their staff identities accurately represent the community they serve. Furthermore, more niche areas of adoption accessibility highlighted by the study participants to consider when designing programs for equity and impact include, but are not limited to, expanded adoption hours and days, temporary adoption events at other locations in the community, improved physical accessibility of the shelter, and instituting a mix of “first come, first served” and set appointment times for people who are not able to wait extended periods of time at the shelter and need a set appointment [51]. Study participants noted that while “first come, first served” policies are fair, they are not always equitable in practice.

## 5. Limitations

Several limitations should be considered alongside these findings. First, the sample size of the study was small (30 participants) and was predominantly comprised of individuals who had successfully adopted an animal from WHS (23 participants), with fewer individuals having adopted and then returned an animal and/or going through the adoption process but ultimately not adopting (6 and 4 participants, respectively). These numbers, as well as the fact that interviews were only conducted with patrons of a single shelter, should be taken into account when considering the representativeness of these data across animal sheltering and rescue more broadly. In addition, a degree of selection bias is present in that qualified participants were people who volunteered and had previously interacted with WHS in some way. Community members deterred from approaching the shelter for one or more reasons are therefore absent. More research should be conducted to confirm the experience of conversation-based animal welfare policies across different organization types that are more nationally representative.

## 6. Conclusions

This study has improved our understanding of how adopters experience conversation-based policies, how these policies affect communities, and how they could be further developed to increase access to the human–animal bond through adoption. Given this study’s findings and previous research, animal shelters should seriously consider implementing open adoption practices and policies. Research is consistently finding that shifting to these policies does not misalign with the goals and mission of the animal welfare and animal protection movement, as equitable access that increases adoptions is not just good for humans in the community served, it is good for the animals in their care.

## Figures and Tables

**Table 1:** Interview participant demographics.

Participant Demographics			
Variable	Value	Frequency	Percentage
Cohort	Adopted	23	77%
	Adopted/Returned	6	20%
	Did Not Adopt	4	13%
Animal	Dog	12	40%
	Cat	21	70%
	Other	1	3%
Gender	Female	19	63%
	Male	8	27%
	Non-Binary	3	10%
Race	Black	2	7%
	Asian	2	7%
	White	23	77%
	Hispanic	4	13%
	American Indian	1	3%
Age Group	18–30	9	30%
	31–45	6	20%
	46–64	8	27%
	65 or older	7	23%
Housing Status	Renter	17	57%
	Homeowner	11	37%
	Temporary Housing	1	3%
	Other	1	3%
Disability	Yes	12	40%
	No	17	57%
	Prefer Not to Answer	1	3%
Disability Type	Invisible	9	30%
	Visible & Invisible	3	10%
	Visible	0	0%

## Data Availability

The raw data supporting the conclusions of this article will be made available upon request of the author(s).

## Appendix A. Interview Guide

1. Why did you choose the Wisconsin Humane Society when trying to adopt an animal?
2. Please describe the process you went through when trying to adopt an animal from the Wisconsin Humane Society. What did you see as the strengths of that process, and what made the process challenging?
3. What information did the adoption team provide about the animal(s) you were interested in adopting?
4. Was there anything that the adoption staff did not share about the animal(s) that you feel should have been added?
5. Did you experience any unfair or disrespectful treatment from the adoption staff? If so, can you tell us what that was like and how that made you feel?
6. Did you experience any particularly fair or respectful treatment from the adoption staff? If so, can you tell us what that was like and how that made you feel?
7. We know the WHS adoption staff may have shared a lot of information with you and asked you many questions. Were there any questions you didn’t feel comfortable answering, information you didn’t feel comfortable sharing, or information you didn’t feel you could be honest about?
8. Do you feel that any adoption policies, practices, or requirements at the Wisconsin Humane Society positively impact individuals in your community trying to adopt an animal? If yes, please provide specific examples.
9. Do you feel that any adoption policies, practices, or requirements at the Wisconsin Humane Society negatively impact individuals in your community trying to adopt an animal? If yes, please provide specific examples.
10. What, if any, policies, practices, or requirements should be changed, added, or taken away to ensure a more fair or equitable adoption experience for yourself or future community members?
11. *“Did not adopt” cohort only:* You shared with us that you went through the process of attempting to adopt an animal from WHS. Could you tell us more about that experience and why you did not ultimately adopt an animal?
12. *“Adopted and returned” cohort only:* You shared with us that you adopted an animal and then returned them to WHS. Could you tell us what the experience of returning an animal was like? Please describe anything that may have helped you keep your animal.
13. Have you ever adopted or acquired an animal from another shelter, rescue, breeder, pet store, family/friend/neighbor, the Internet, or other source? If so, can you tell us about that process? How did it differ from your experience at Wisconsin Humane Society?
14. This study aims to understand the strengths and challenges of current approaches in open-adoption or low-barrier adoption policies and practices from the perspective of community members. What did we not ask about that you think is important to share?
